# Association of Physical Activity in Midlife With Mobility Limitations and Falls in Old Age—A Longitudinal Twin Study

**DOI:** 10.1177/08982643251395871

**Published:** 2025-11-12

**Authors:** Pia Pullinen, Jari Parkkari, Jaakko Kaprio, Elina Sillanpää, Harri Sievänen, Urho Kujala, Katja Waller

**Affiliations:** 1 Faculty of Sport and Health Sciences, 4168 University of Jyväskylä, Finland; 2 Faculty of Medicine and Health Technology, 7840 University of Tampere, Finland; 3 Institute for Molecular Medicine Finland (FIMM), University of Helsinki, Finland; 4 The Wellbeing Services County of Central Finland, Jyväskylä, Finland; 5 The UKK Institute for Health Promotion Research, Tampere, Finland

**Keywords:** middle age, physical activity, mobility limitations, falls, twins

## Abstract

**Objective:**

To study the associations between midlife physical activity and later-life mobility limitation and falls in monozygotic and dizygotic twin pairs.

**Methods:**

Midlife physical activity data from 1975, 1981, and 1990 questionnaires were drawn from the Older Finnish Twin Cohort. In the third survey, the participants were aged 46–50 years. Mobility limitations (none, some or severe) and falls (no falls, single fall or recurrent falls) were self-reported 2014–2016. Data (*n* = 641) were analyzed using multinomial logistic regression models.

**Results:**

In individual-level analysis a low level of physical activity in midlife was associated with severe mobility limitations. In within-twin pair analyses physical activity in midlife was associated with single falls among all pairs.

**Discussion:**

Lower levels of self-reported physical activity during midlife were associated with severe mobility limitations over two decades later. However, this association was attenuated when controlled for genetic factors.

## Introduction

The increasing number and proportion of older adults in the general population is well-documented. Aging elevates the risk of developing various diseases (Christensen et al., 2009), increases mortality (Cheng et al., 2020; Hardy et al., 2011), and contributes to a higher risk of mobility limitations (Heiland et al., 2016) and falls (World Health Organization, 2008).

Mobility is crucial for functioning well and maintaining physical independence (Hirvensalo et al., 2000). Approximately 30% of older adults aged 60 and above experience mobility limitations, with the majority of individuals over 80 years facing such challenges (Musich et al., 2018; Wang et al., 2024). Enhanced mobility reduces the need for healthcare services (Hardy et al., 2011; Musich et al., 2018) and is associated with lower mortality risk (Hirvensalo et al., 2000; Wang et al., 2024) and further disability (Heiland et al., 2016). Mobility limitations have also been found to be a risk factor for falls (Wang et al., 2024). In this study, mobility was described as the ability to walk and climb stairs both safely and independently, following the definition by Guralnik et al. (2000).

Falls, a major cause of mortality, place a significant burden on healthcare systems (World Health Organization, 2021). One in three community-dwelling older adults aged over 65 experiences at least one fall each year (Tinetti & Kumar, 2010). Falls often lead to a fear of falling, causing older adults to limit their daily activities, further impairing their functional capacity and increasing the risk of future falls (Schoene et al., 2019).

To successfully reduce mobility limitations and falls in older adults, it is essential to understand their underlying causes and risks. Life course studies suggest that numerous difficulties experienced in old age may originate from earlier life stages (Atallah et al., 2018; Sabia et al., 2012). Middle age is a long period during which lifestyle choices can affect functional ability in later years. Physical activity is one of these potentially modifiable lifestyle choices that have preventative potential (Lafortune et al., 2016). Physical activity plays an important role in maintaining mobility (Morie et al., 2010; Visser et al., 2005) and preventing falls among older adults (Lee, 2020; Sherrington et al., 2020; Vieira et al., 2016). Studies show an association between higher levels of physical activity during midlife and better mobility later in life (Artaud et al., 2016; Chang et al., 2013; Malmberg et al., 2006; Patel et al., 2006; Pettee Gabriel et al., 2017; Tikkanen et al., 2012). Physical activity in midlife has also been found to reduce the risk of falls in older age (Gabriel et al., 2019; Smith et al., 2019). However, longitudinal studies in which physical activity data are collected in midlife and the follow-up time is over 20 years are limited. Hence, to determine if physical activity is potentially preventative requires longer follow-up studies with data collected in midlife.

Twin studies offer a unique opportunity to unravel genetic and environmental influences on health outcomes in later life. Monozygotic twins share all of their genes at the sequence level, whereas dizygotic twins share on average 50% of their segregating genes (as do full siblings). In the classical twin study design, the similarity of health outcomes is compared in these two groups and thus allows estimation of the relative contributions of genetic and environmental effects on the total variability of the trait under study. Such estimations of genetic and environmental variance can be extended to multivariate designs, to examine multiple traits or the same trait over time longitudinally (Hagenbeek et al., 2023). When studying the association between an exposure and outcome, within-pair analyses compare differences between twins in a pair, thereby controlling for shared familial factors; persistence of an association within pairs supports a potential causal effect, whereas attenuation suggests confounding by genetics or shared environment.

Observational studies in unrelated individuals cannot conclusively distinguish between a causal relationship from exposure to outcome and confounding; confounding variables can often be measured and accounted for, but unmeasured confounding can remain. Pleiotropy, which results from genes affecting both the exposure and outcome, is a potential source of unmeasured confounding. Co-twin control designs, where exposure-discordant twin pairs are used, further address confounding by familial factors such as shared genes and environment. Using siblings or DZ twins as controls automatically accounts for unmeasured family-level confounders, and in MZ pairs, this approach also fully controls for genetic confounding. If an exposure truly causes an outcome, the association should be observed both at the individual level and within-pairs; attenuation of the association in DZ and especially in MZ twins indicates confounding by genetics or the shared environment (McGue et al., 2010).

Genetic predispositions may influence both the ability and motivation to engage in physical activity (de Geus, 2023), as well as underlying physiological traits such as muscle strength, which are associated with better mobility (Herranen et al., 2024). Previous twin studies have shown that physical activity in midlife can reduce the risk of chronic diseases (Waller, Kaprio, & Kujala, 2008), is linked to lower mortality in older age (Waller et al., 2010) and appears to have beneficial effects on several health indicators such as blood values and waist circumference (Kujala et al., 2022). However, little is known about whether the relationship between midlife physical activity and later-life mobility limitations is independent of genetic factors.

The main aim of this study was to examine associations between self-reported longitudinal physical activity in adulthood and mobility limitations and falls in long-term, that is, 24 years later. Our hypothesis was that individuals who were physically active in midlife would have fewer mobility limitations and falls in older age compared to those who were less active in midlife. We also hypothesized that shared genetic factors may partially explain this association.

## Methods

### Study Participants

The study is based on the Older Finnish Twin Cohort, which consists of same-sex twin pairs (13 888 pairs) born in Finland before 1958 and with both co-twins alive in 1975 (Kaprio & Koskenvuo, 2002). Data were collected in four waves. In 1975, a health questionnaire, including questions on physical activity, was sent to all twin candidates. The next questionnaire, mailed in 1981, was sent to all twins who were alive and resident in Finland at that time. In 1990, a third questionnaire was sent to participants who had responded to either of the earlier questionnaires. Questionnaire data from 1975, 1981, and 1990 correspond to waves 1, 2, and 3, respectively and wave 4 refers to the MOBILETWIN study conducted later. The response rates for the three questionnaires were 88%, 91%, and 77% (Romanov et al., 2003; Waller et al., 2014).

Later, the twin pairs born between 1940 and 1944 were invited to participate in the physical activity and physical functioning study (MOBILETWIN) conducted between 2014 and 2016. The physical functioning questionnaire was sent only to those twin individuals who had responded to at least one of the earlier questionnaires (1975, 1981 or 1990). The sample size was 3186 twin individuals, of whom 1632 twin individuals (816 complete twin pairs) were both alive and contactable. The inclusion and exclusion criteria have been detailed earlier (Waller et al., 2018).

The final cohort comprised those who had responded to the questionnaires in 1975, 1981, and 1990 and had completed the physical functioning questionnaire as part of the MOBILETWIN study (*n* = 641 individuals of whom 301 (47%) were men and 340 (53%) women). The mean age of the participants at follow-up was 73 years (range 71–75). The study group consisted of 205 complete pairs, of whom 91 were monozygotic, 102 dizygotic, and 12 of unknown zygosity.

### Measurement of Leisure-time Physical Activity

Data on physical activity were collected by questionnaires. The questions in 1975 and 1981 were similar, whereas the 1990 questionnaire differed slightly from the previous ones. In 1975 and 1981, physical activity was assessed using multiple items covering leisure-time physical activity (frequency per month, duration per session, and intensity) as well as commuting physical activity. In the 1990 questionnaire, the same information was obtained using a single question. The question on the intensity of physical activity was formulated as follows: Is your physical activity about as strenuous on average as (1) walking, (2) alternately walking and jogging, (3) jogging (light running), or (4) running? Participants were also asked how much time they spent daily on actively commuting to and from work. The questionnaires did not specify the types of physical activity but covered all physical activity occurring in daily life. Based on the responses, an MET index (MET h/day) was calculated. The index was calculated by assigning a MET score to each activity and by calculating the product of that activity: intensity x duration x frequency at each time point, and then converted into MET h/day (Kujala et al., 1998). This questionnaire-based MET index has been found to correlate well with the MET index obtained from a detailed interview (Waller, Kaprio, & Kujala, 2008). To determine the overall volume of physical activity at baseline, mean MET index was calculated. This was done by averaging the annual MET index values from the three baseline years. Collecting physical activity data from multiple time points and averaging the results reduces the influence of temporary fluctuations. This approach provides a more stable and representative estimate of habitual physical activity over time and has therefore been regarded as a methodological strength in previous study (Waller et al., 2018). In 1975, the MET index was 2.22 MET h/day, in 1981 2.50 MET h/day, and in 1990 3.27 MET h/day.

### Self-Reported Mobility Limitations and Falls

Data on mobility limitations and falls were collected in the MOBILETWIN study between 2014 and 2016 by a structured questionnaire. Participants were asked if they experienced problems in performing three different tasks: walking 2 km, walking 0.5 km, or climbing up one flight of stairs. These questions were structured as follows: “Do you have difficulty in…?” and five alternative given answers were 1 = able to manage without difficulty, 2 = able to manage with some difficulty, 3 = able to manage with a great deal of difficulty, 4 = able to manage but only with help of someone, and 5 = unable to manage even with the help of another person.

In line with previously established categories (Mänty et al., 2007), mobility difficulties were classified into three groups: 1 = No mobility limitations in any of three tasks (able to manage without difficulty); 2 = Some mobility limitations in at least one of the three tasks (able to manage with some difficulty); and 3 = Severe mobility limitations or unable to perform any of the three tasks (able to manage with a great deal of difficulty, able to manage but only with help of another person, unable to manage even with help of another person). If a participant reported no difficulty walking 2 km and the 0.5 km walking information was missing, the 0.5 km variable was coded as ‘no difficulty’. The analyses included participants who had available data on all three mobility items: walking 2 km, walking 0.5 km, and climbing stairs.

Falls were inquired about by asking participants if they had fallen indoors, outdoors in a yard or at a public place during the past 12 months and if so, how many times. Participants were classified as non-fallers, single fallers if they reported one fall, and as recurrent fallers if they reported more than one fall (Jehu & Skelton, 2023).

### Background Characteristics

Participants’ background data was also collected by questionnaires. Body mass index (BMI) was calculated using self-reported weight and height. Smoking status was classified into four groups: never, occasional, former, and current. Alcohol consumption was assessed through multiple questions and expressed as grams of absolute alcohol per day (Piirtola et al., 2016). Education and social class were reclassified into three categories: white collar, blue collar and gray collar. The white-collar group comprised participants who had at least 12 years of education and performed sedentary work. The blue-collar group had less than 9 years of education and jobs involving walking, lifting, or hard physical labor. Those who did not meet either of these sets of criteria formed the gray-collar group (Romanov et al., 2003).

### Statistical Analyses

Data analyses were conducted using IBM SPSS Statistics version 26 and Stata IC version 16. Participants’ characteristics were described using means and standard deviation (SD) or numbers and percentages.

In the individual-level analysis, in which the twins were treated as individuals, multinomial logistic regression models were used. As twins within pairs are not independent observations due to sampling of pairs, a primary sampling unit (PSU) was applied to account for within-family correlation. The analysis only included participants with MET values from all 3 years (*n* = 641). A fixed-effects multinomial logistic model was used in the within-twin pair analysis, which was conducted for all pairs together (monozygotic, dizygotic, and unknown zygosity combined).

Two models were used in both the individual and within-twin pair analyses: model 1, adjusted for age and sex; and model 2, further adjusted for BMI in 1990. In the mobility limitations analysis, the reference group had no mobility limitations, and in the falls analysis, the reference group was non-fallers. McNemar’s test was used in additional analyses when analyzing pairwise differences among twin pairs discordant for self-reported physical activity. Discordance was defined as one twin having a mean MET index of ≥2 and the co-twin having a mean MET index of <2 (Waller et al., 2008). In the analyses of discordant pairs, mobility limitation and falls were treated as dichotomous variables (No mobility limitations vs. Mobility limitations/Non-faller vs. Faller).

## Results

The basic characteristics of the study sample by mobility limitations are shown in Table 1. Of the 641 participants, 495 (77%) reported not having mobility limitations, 103 (16%) reported having some problems, and 43 (7%) reported having severe problems in walking 2 km and/or 0.5 km and/or climbing up one flight of stairs. In 1990, the group with no mobility limitations had lower BMI, fewer smokers and lower alcohol use than the other groups. The same group also contained a higher proportion of white-collar participants.

**Table 1.** Characteristics of study cohort in 1990 by mobility limitation level

Variable	No mobility limitations *n* = 495	Some mobility limitations *n* = 103	Severe mobility limitations *n* = 43
Sex, *n* (%)			
Men	238 (79)	44 (15)	19 (6)
Women	257 (76)	59 (17)	24 (7)
Age (y), mean (SD)	47.9 (1.4)	47.9 (1.4)	47.9 (1.6)
BMI (kg/m^2^), mean (SD)	24.1 (2.8)	26.7 (3.8)	27.7 (4.9)
Smoking, *n* (%)			
Never	283 (58)	51 (50)	22 (51)
Occasional	18 (4)	6 (6)	0 (0)
Former	118 (24)	24 (23)	11 (26)
Current	72 (15)	22 (21)	10 (23)
Alcohol use (g/day), mean (SD)	8.66 (10.8)	8.72 (12.2)	10.82 (13.8)
Education and social class *n* (%)			
Blue-collar	157 (32)	45 (45)	23 (54)
Gray-collar	235 (48)	51 (51)	17 (40)
White-collar	94 (19)	5 (5)	3 (7)
Past physical activity			
MET index 1975, mean (SD)	2.30 (2.61)	2.17 (2.25)	1.39 (1.46)
MET index 1981, mean (SD)	2.58 (2.74)	2.38 (2.55)	1.88 (1.97)
MET index 1990, mean (SD)	3.44 (3.25)	2.97 (2.75)	2.11 (2.05)
Mean MET index, mean (SD)	2.77 (2.18)	2.51 (1.87)	1.80 (1.46)
Non-fallers and fallers at follow-up, *n* (%)			
Non-faller	430 (87)	85 (83)	31 (72)
Single faller	38 (8)	5 (5)	5 (12)
Recurrent faller	27 (6)	13 (13)	7 (16)

Note. SD = standard deviation; BMI = body mass index; MET = metabolic equivalent. Education and social class data are from 1981.

At least one fall was reported by 95 (15%) of the 641 participants, of whom 47 were recurrent fallers. Women accounted for 61% of all fallers. In total, 58 women, representing 17% of all the participating women, reported falls. Altogether, 65 fallers (68%) did not report mobility limitations, 18 fallers (19%) reported some mobility limitations, and 12 fallers (13%) reported severe mobility limitations.

### Physical Activity and Mobility Limitation: Individual-Level Analyses

In the individual-level multinomial logistic regression model of mean MET index in midlife and mobility limitation at follow-up (Table 2), the participants who had a higher mean MET index had fewer mobility limitations than the participants who had a lower mean MET index. The baseline mean MET index was 2.77 (SD = 2.18) for those participants who reported no mobility limitations at follow-up, 2.51 (SD = 1.87) for those reporting some mobility limitations, and 1.80 (SD = 1.46) for those reporting severe mobility limitations. In the regression analysis (model 1), each 1 mean MET index increase in physical activity was associated with a 31% lower risk of being in the severe mobility limitation group compared to the no mobility limitation group (RRR = 0.69, 95% CI 0.51–0.93). The association between mean MET index and some mobility limitations was not statistically significant (RRR = 0.94, 95% CI 0.84–1.06). These findings persisted after adjusting for BMI (1990). Further adjustment for education and social class (1981), smoking status (1990), and alcohol use (1990), the RRR remained essentially the same as in model 2 (results not shown).

**Table 2.** Multinomial logistic regression of mobility limitation by physical activity

Mean MET index	Individual-level analyses	Within-twin pair analyses
	(*n* = 641)	All (*n* = 205 pairs)*
	RRR (95% CI)	RRR (95% CI)
meanMET (model 1)		
No mobility limitation	Ref	Ref
Some mobility limitation	0.94 (0.84, 1.06)	1.05 (0.82, 1.33)
Severe mobility limitation	**0.69 (0.51, 0.93)**	0.48 (0.17, 1.30)
meanMET (model 2)		
No mobility limitation	Ref	Ref
Some mobility limitation	0.98 (0.88, 1.09)	1.07 (0.83, 1.37)
Severe mobility limitation	**0.73 (0.54, 0.99)**	0.32 (0.08, 1.25)

Note. MET = metabolic equivalent. All = Dizygotic twin pairs, monozygotic twin pairs and unknown zygotic twin pairs altogether. There are 12 unknown zygosity twin pairs. Model 1 is adjusted for sex and age in 1990. Model 2 is adjusted for sex, age in 1990 and BMI in 1990. In within-twin pair analyses, sex and age are not included as covariates because twins share the same sex and age. The results written in bold are statistically significant (*p* < .05). *55 pairs were informative and included in the analyses.

### Physical Activity and Mobility Limitation: Within-Twin Pair Analyses

In the within-twin pair analyses, in model 1, no statistically significant association was found between mean MET index and some mobility limitations (RRR = 1.05, 95% CI 0.82–1.33). For severe mobility limitations, higher physical activity was associated with a substantially lower relative risk (RRR = 0.48, 95% CI 0.17–1.30), corresponding to a 52% reduction for each one-point increase in the mean MET index, although this association did not reach statistical significance. These findings persisted after adjusting for BMI (1990). As in individual analyses, further adjustment for covariates, education and social class (1981), smoking status (1990), and alcohol use (1990), the RRR remained essentially the same as in model 2. Within-twin pair analyses were conducted only for models 1 and 2, for all pairs where sample size was sufficient (Table 2). Models for MZ pairs alone could not be analyzed due to convergence issues, indicating a high degree of resemblance of MZ twins and insufficient differences for analysis.

### Physical Activity and Falls: Individual-Level Analyses

In the individual-based multinomial logistic regression model of mean MET index in midlife and falls at follow-up (Table 3), the participants with a higher mean MET index reported fewer single falls than those with a lower mean MET index. The baseline mean MET index was 2.69 (SD = 2.15) for those participants reporting no falls at follow-up, 2.17 (SD = 1.38) for those participants reporting single falls, and 2.81 (SD = 2.18) for those reporting more than one fall. In the regression analysis (model 1), each 1 mean MET index increase in physical activity is associated with a 14% lower relative risk of being in the single faller group compared to the non-faller group (RRR = 0.86, 95% CI 0.73–1.02) and each 1 mean MET index increase in physical activity was associated with a 3% higher relative risk of being in the recurrent faller group compared to the non-faller group (RRR = 1.03, 95% CI 0.90–1.19). The associations between the mean MET index and single fallers and refallers were not statistically significant. These findings persisted after adjusting further for BMI (1990). Further adjustment for education and social class (1981), smoking status (1990), and alcohol use (1990), the RRR remained essentially the same as in model 2 (results not shown).

**Table 3.** Multinomial logistic regression of falls by physical activity

Mean MET index	Individual-level analyses	Within-twin pair analyses
	(*n* = 641)	All (*n* = 205 pairs)*
	RRR (95% CI)	RRR (95% CI)
meanMET (model 1)		
Non-faller	Ref	Ref
Single faller	0.86 (0.73, 1.02)	**0.67 (0.45, 1.00)**
Recurrent faller	1.03 (0.90,1.19)	1.03 (0.75, 1.43)
meanMET (model 2)		
Non-faller	Ref	Ref
Single faller	0.87 (0.74, 1.02)	0.68 (0.44, 1.04)
Recurrent faller	1.05 (0.91, 1.21)	1.03 (0.75, 1.43)

Note. MET = metabolic equivalent. All = Dizygotic twin pairs, monozygotic twin pairs and unknown zygotic twin pairs altogether. There are 12 unknown zygosity twin pairs. Model 1 is adjusted for sex and age in 1990. Model 2 is adjusted for sex, age in 1990 and BMI in 1990. In within-twin pair analyses, sex and age are not included as covariates because twins share the same sex and age. The results written in bold are statistically significant (*p* < .05). *44 pairs were informative and included in the analyses.

### Physical Activity and Falls: Within-Twin Pair Analyses

In the within-twin pair analyses, in model 1, a statistically significant association was observed between mean MET index and the single faller group (RRR = 0.67, 95% CI 0.45–1.00) but not between mean MET index and the recurrent faller group (RRR = 1.03, 95% CI 0.75–1.43). In model 2, statistically significant associations were not seen, although the results were similar. As in individual analyses, further adjustment for covariates, education and social class (1981), smoking status (1990) and alcohol use (1990), the RRR remained essentially the same as in model 2. Within-twin pair analyses were conducted only for models 1 and 2, for all pairs where sample size was sufficient (Table 3). Models for MZ pairs alone could not be analyzed due to convergence issues, indicating a high degree of resemblance of MZ twins and insufficient differences for analysis.

### Discordant for Self-Reported Physical Activity: Pairwise Analyses

Analyses among 83 twin pairs discordant for self-reported physical activity related to mobility limitations did not reveal any statistically significant results (Table 4). Similarly, no significant results were observed for discordance in self-reported physical activity related to falls (Table 5).

**Table 4.** Mobility limitation in twin pairs discordant for physical activity

	Higher mean MET index co-twins	Lower mean MET index co-twins	*p*-value
All pairs *n* = 83			
No mobility limitations, *n* (%)	68 (82)	65 (78)	.678
Mobility limitations, *n* (%)	15 (18)	18 (22)	
DZ pairs (*n* = 43 pairs)			
No mobility limitations, *n* (%)	36 (84)	32 (74)	.454
Mobility limitations, *n* (%)	7 (16)	11 (26)	
MZ pairs (*n* = 35 pairs)			
No mobility limitations, *n* (%)	28 (80)	29 (83)	1.000
Mobility limitations, *n* (%)	7 (20)	6 (17)	

Note. MET = metabolic equivalent; DZ = dizygotic twin; MZ = monozygotic twin. All = Dizygotic twin pairs, monozygotic twin pairs and unknown zygotic twin pairs altogether. There are 5 unknown zygosity twin pairs. According to McNemar’s test. Higher mean MET co-twin: mean MET index ≥2. Lower mean MET co-twin: mean MET <2.

**Table 5.** Falls in twin pairs discordant for physical activity

	Higher mean MET index co-twins	Lower mean MET index co-twins	*p*-value
All pairs (*n* = 83)			
Non-faller, *n* (%)	72 (87)	69 (83)	.648
Faller, *n* (%)	11 (13)	14 (17)	
DZ pairs (*n* = 43 pairs)			
Non-faller, *n* (%)	36 (84)	35 (81)	1.000
Faller, *n* (%)	7 (16)	8 (19)	
MZ pairs (*n* = 35 pairs)			
Non-faller, *n* (%)	32 (91)	30 (86)	.727
Faller, *n* (%)	3 (9)	5 (14)	

Note. MET = metabolic equivalent; DZ = dizygotic twin; MZ = monozygotic twin. All = Dizygotic twin pairs, monozygotic twin pairs and unknown zygotic twin pairs altogether. There are 5 unknown zygosity twin pairs. According to McNemar’s test. Higher mean MET co-twin: mean MET index ≥2. Lower mean MET co-twin: mean MET <2.

## Discussion

This longitudinal study of Finnish community-dwelling older twins in their 70 s indicates that self-reported physical activity in midlife, gathered during a 15-year period, is associated with severe mobility limitations. Participants who reported higher physical activity also reported fewer mobility limitations and single falling during the preceding year than those reporting lower physical activity. However, in the within-twin pair analyses, comparing each twin with their co-twin, the associations between self-reported physical activity in midlife and mobility limitations weakened when genetic factors were controlled for and did not reach statistical significance, suggesting that the association may be influenced by genetic factors. In contrast, in the within-twin pair analysis, the association between self-reported physical activity in midlife and falls was statistically significant, suggesting that it may be independent of shared genetic and early-life environmental factors.

Our results support findings in previous studies on midlife physical activity and mobility limitations. Being physically active during midlife is associated with better mobility in old age (Artaud et al., 2016; Chang et al., 2013; Malmberg et al., 2006; Patel et al., 2006; Pettee Gabriel et al., 2017; Tikkanen et al., 2012). Patel et al. (2006) and Tikkanen et al. (2012) investigated whether self-reported physical activity in midlife, recalled retrospectively, was associated with mobility in old age. They found that older adults who reported higher levels of physical activity in midlife had better mobility in later life than those who were less physically active. Chang et al. (2013), who examined the long-term association between self-reported midlife physical activity and measured physical function in later-life, concluded that engaging in physical activity during midlife is important for maintaining physical function in later years. Artaud et al. (2016) and Pettee Gabriel et al. (2017) used physical activity trajectories to examine the long-term association between self-reported midlife physical activity and mobility-related outcomes. Both studies also found that midlife engagement in physical activity is important for maintaining physical function in later life. Artaud et al. (2016) collected mobility data through questionnaires, whereas Pettee Gabriel et al. (2017) utilized objective measurements. Malmberg et al. (2006) assessed the associations of midlife physical activity with the risk of impaired mobility in later life. They found an association between self-reported low physical activity in midlife and increased future risk of self-reported mobility impairment. Physical activity was classified into three leisure time physical activity categories: low, moderate and high. In our study, data on physical activity were collected at the time of the midlife questionnaires and converted into an MET index (MET h/day). Data on mobility limitations were elicited by a questionnaire and grouped into three categories.

Although our results on the association between midlife physical activity and falls are not statistically significant, they are in line with previous evidence. Being physically active in midlife and beyond is associated with fewer falls in old age (Kwok et al., 2024; Pettee Gabriel et al., 2019; Smith et al., 2019). Kwok et al. (2024) explored the relationship between long-term physical activity participation and falls among women. Their data on baseline physical activity were collected by a questionnaire administered at seven time points. They found that individuals who reported consistently low levels of physical activity over a period of 18 years had an increased risk of subsequent self-reported falls. Pettee Gabriel et al. (2019) analyzed the relationship between self-reported moderate to vigorous physical activity trends during midlife and information on falls obtained by telephone interviews 25 years later. Their results suggest that physical activity from midlife to older adulthood can reduce fall risk. Smith et al. (2019) investigated changes in physical activity behavior and risk of falls. They found that, compared to being physically inactive, maintaining physical activity over a 4-year period was associated with a reduced risk of falls. In our study, an increase in physical activity was associated with a lower risk of being in the single faller group. Higher physical activity was associated with an increased risk of being in the refaller group. However, when we examined the distribution of refallers across tertiles of the mean MET index among the refallers, only three participants belonged to the highest tertile (7.60–11.21), whereas the majority (*n* = 39) belonged to the lowest tertile (0.37–3.98). This indicates that the apparent association should be interpreted cautiously, given the small number of participants in the highest activity tertile.

Previous studies have shown that around 30% of older adults aged 65 and over experience mobility difficulties (Musich et al., 2018) and that approximately 30% of community-dwelling individuals aged 65 and older fall each year (Gillespie et al., 2012). In our study, 23% of the participants had difficulties in walking 2 km and/or 0.5 km and/or climbing up one flight of stairs, and only 15% of participants had fallen during the preceding year. Our participants were relatively young community-dwelling older men and women who probably had relatively good functional ability. However, we cannot be certain because physical performance was not measured at follow-up.

The previous research has mainly focused on individuals, whereas our sample comprised twins, allowing us to assess the effect of genetics and childhood environment in addition to conducting analyses on individual twins. Unlike the individual-level analyses, no significant associations between physical activity and mobility limitations were found in the within-twin pair analyses. This suggests that these associations are probably affected by genetic and early-life environmental factors. However, the within-twin pair analyses indicated a stronger association between physical activity and severe mobility limitations compared with the individual-level analyses, thereby implying the opposite. The associations between physical activity and falls in the within-twin pair analyses in all pairs were similar to those obtained in individual-level analysis. However, this was not confirmed in model 1, where an association was observed between the mean MET index and the single faller group, indicating that the associations might not be affected by genetic factors. In pairs discordant for self-reported physical activity, the association between physical activity and mobility limitations and falls did not appear to be independent of genetics and early-life factors. Because the number of twin pairs was limited both in the within-twin pair analyses and in the analyses of discordant pairs, the results may be subject to uncertainty. Therefore, we cannot draw firm conclusions about the influence of genetic factors and childhood environment on the outcomes, although there are indications of such effects.

### Limitations and Strengths

Our study has its limitations. The baseline physical activity data were gathered using self-reports. At the time the baseline questionnaires were administered, objective tools to measure physical activity were unavailable. The questionnaires did not differentiate between types of physical activity, so the specific nature of the reported activities is unknown. Nevertheless, it is reasonable to assume that resistance training was less common at the time, particularly in the earlier surveys, and that most activity was aerobic in nature. However, the physical activity questionnaires utilized in this study can be considered valid (Iso-Markku et al., 2016; Piirtola et al., 2016; Waller et al., 2010). The information on falls was collected through a questionnaire in which participants were asked whether they had experienced a fall during the previous 12 months. This may have led to inaccuracies in reporting falls (Garcia et al., 2015), although a one-year recall period is considered acceptable in collecting fall data (Ganz et al., 2005). The rather small number of fallers, especially recurrent fallers, may have reduced the statistical power of some of our analyses. A further limitation of the study is that we did not have information on individuals with mobility limitations at the 1990 baseline and thus could not exclude them from the study. However, the number of such participants would likely have been small (Koskinen et al., 2012). Moreover, the relatively long follow-up period allows for the possibility of changes in post-baseline physical activity levels, for instance, due to illness, potentially influencing the results. However, engaging in physical activity during midlife raises the likelihood of remaining active in old age (Aggio et al., 2017). The long follow-up period can also be seen as a strength, as we wanted to examine the relationship between midlife physical activity and mobility limitations and falls in older age. Another strength is the twin study design, which enabled us to appraise genetic influences. The number of pairs who differ in the exposures and outcomes are relatively small, hence the power of the twin design to provide evidence for or against causal association is limited (Gustavson et al., 2024). A further strength of this study is the collection of physical activity data at three different time points (1975, 1981 and 1990), which provides a more accurate representation of midlife physical activity. On the other hand, requiring data on physical activity from all 3 years has resulted in a reduced sample size. In the final sample, participants had healthier lifestyles: smoking and alcohol consumption were less common than among the participants who were excluded from the analyses. In the final sample, participants also had, on average, higher education and a more favorable social class. Self-reported mobility limitations have been shown to be a valid method of assessing mobility (Brown & Flood, 2013). The current questionnaire asked about respondents’ ability to perform three mobility tasks: walking 2 km, walking 0.5 km, and climbing a single flight of stairs. This structured questionnaire has been demonstrated to provide a valid assessment of mobility (Mänty et al., 2007). In summary, beyond the finding that lower self-reported physical activity in midlife predicts severe mobility limitations, the novelty of our study lies in repeated activity assessments, exceptionally long-term follow-up, and the use of a large, nationally representative twin cohort, enabling a unique examination of genetic and environmental influences on later-life mobility and falls.

## Conclusion

This study provides longitudinal evidence of an association between a low level of midlife physical activity and a higher prevalence of self-reported mobility limitation later in life among community-dwelling older adults aged 72 and over. Personalized physical activity guidance and programs should be offered to middle-aged individuals with low physical activity levels to support their functional ability and reduce their risk of falls later in life. Future research is needed to examine the amount and intensity of physical activity sufficient to maintain the functional ability of aging people over a decade. In addition, studies using larger twin samples are warranted to better assess the role of genetic factors and childhood environment in the associations between physical activity, mobility limitations, and falls. Since the association between physical activity and severe mobility limitation was not evident within twin pairs, future research should further explore the potential genetic mechanisms underlying this relationship.

## Data Availability

Because of the consent given by the study participants and the high degree of identifiability of the twin siblings in Finland, the data cannot be made publicly available.*

## References

[bibr1-08982643251395871] Aggio D. Papacosta O. Lennon L. Whincup P. Wannamethee G. Jefferis B. J. (2017). Association between physical activity levels in mid-life with physical activity in old age: A 20-year tracking study in a prospective cohort. BMJ Open, 7(8), Article e017378. 10.1136/bmjopen-2017-017378 PMC5724234 28935690

[bibr2-08982643251395871] Artaud F. Sabia S. Dugravot A. Kivimaki M. Singh-Manoux A. Elbaz A. (2016). Trajectories of unhealthy behaviors in midlife and risk of disability at older ages in the whitehall II cohort study. The Journals of Gerontology. Series A, Biological Sciences and Medical Sciences, 71(11), 1500–1506. 10.1093/gerona/glw060 PMC5055647 27034508

[bibr3-08982643251395871] Atallah N. Adjibade M. Lelong H. Hercberg S. Galan P. Assmann K. E. Kesse-Guyot E. (2018). How healthy lifestyle factors at midlife relate to healthy aging. Nutrients, 10(7), 854. 10.3390/nu10070854 PMC6073192 29966372

[bibr4-08982643251395871] Brown C. J. Flood K. L. (2013). Mobility limitation in the older patient: A clinical review. JAMA, 310(11), 1168–1177. 10.1001/jama.2013.276566 24045741

[bibr5-08982643251395871] Chang M. Saczynski J. S. Snaedal J. Bjornsson S. Einarsson B. Garcia M. Aspelund T. Siggeirsdottir K. Gudnason V. Launer L. J. Harris T. B. Jonsson P. V. (2013). Mid-life physical activity preserves lower extremity function in older adults: Age gene/environment susceptibility (AGES) - Reykjavik study. Journal of the American Geriatrics Society, 61(2), 237–242. 10.1111/jgs.12077 PMC4205047 23320618

[bibr6-08982643251395871] Cheng X. Yang Y. Schwebel D. C. Liu Z. Li L. Cheng P. Ning P. Hu G. (2020). Population ageing and mortality during 1990–2017: A global decomposition analysis. PLOS Medicine, 17(6), Article e1003138. 10.1371/journal.pmed.1003138 PMC7279585 32511229

[bibr7-08982643251395871] Christensen K. Doblhammer G. Rau R. Vaupel J. W. (2009). Ageing populations: The challenges ahead. Lancet (London, England), 374(9696), 1196–1208. 10.1016/S0140-6736(09)61460-4 PMC2810516 19801098

[bibr8-08982643251395871] de Geus E. J. C. (2023). Genetic pathways underlying individual differences in regular physical activity. Exercise and Sport Sciences Reviews, 51(1), 2–18. 10.1249/JES.0000000000000305 PMC9762726 36044740

[bibr10-08982643251395871] Gabriel K. P. Griswold M. E. Wang W. Conway S. H. Windham B. G. Palta P. Kucharska-Newton A. Pompeii L. A. (2019). Physical activity trajectories and subsequent fall risk: ARIC study. Preventive Medicine, 121(8), 40–46. 10.1016/j.ypmed.2019.02.007 PMC6448408 30742870

[bibr11-08982643251395871] Ganz D. A. Higashi T. Rubenstein L. Z. (2005). Monitoring falls in cohort studies of community-dwelling older people: Effect of the recall interval. Journal of the American Geriatrics Society, 53(12), 2190–2194. 10.1111/j.1532-5415.2005.00509.x 16398908

[bibr12-08982643251395871] Garcia P. A. Dias J. M. D. Silva S. L. A. Dias R. C. (2015). Prospective monitoring and self-report of previous falls among older women at high risk of falls and fractures: A study of comparison and agreement. Brazilian Journal of Physical Therapy, 19(3), 218–226. 10.1590/bjpt-rbf.2014.0095 PMC4518575 26083603

[bibr13-08982643251395871] Gillespie L. D. Robertson M. C. Gillespie W. J. Sherrington C. Gates S. Clemson L. Lamb S. E. (2012). Interventions for preventing falls in older people living in the community. The Cochrane Database of Systematic Reviews, 2012(9), Article CD007146. 10.1002/14651858.CD007146.pub3 PMC8095069 22972103

[bibr14-08982643251395871] Guralnik J. M. Ferrucci L. Pieper C. F. Leveille S. G. Markides K. S. Ostir G. V. Studenski S. Berkman L. F. Wallace R. B. (2000). Lower extremity function and subsequent disability: Consistency across studies, predictive models, and value of gait speed alone compared with the short physical performance battery. The Journals of Gerontology. Series A, Biological Sciences and Medical Sciences, 55(4), M221–M231. 10.1093/gerona/55.4.m221 PMC12149745 10811152

[bibr15-08982643251395871] Gustavson K. Torvik F. A. Davey Smith G. Røysamb E. Eilertsen E. M. (2024). Familial confounding or measurement error? How to interpret findings from sibling and co-twin control studies. European Journal of Epidemiology, 39(6), 587–603. 10.1007/s10654-024-01132-6 PMC11249619 38879863

[bibr16-08982643251395871] Hagenbeek F. A. Hirzinger J. S. Breunig S. Bruins S. Kuznetsov D. V. Schut K. Odintsova V. V. Boomsma D. I. (2023). Maximizing the value of twin studies in health and behaviour. Nature Human Behaviour, 7(6), 849–860. 10.1038/s41562-023-01609-6 37188734

[bibr17-08982643251395871] Hardy S. E. Kang Y. Studenski S. A. Degenholtz H. B. (2011). Ability to walk 1/4 mile predicts subsequent disability, mortality, and health care costs. Journal of General Internal Medicine, 26(2), 130–135. 10.1007/s11606-010-1543-2 PMC3019329 20972641

[bibr18-08982643251395871] Heiland E. G. Welmer A.-K. Wang R. Santoni G. Angleman S. Fratiglioni L. Qiu C. (2016). Association of mobility limitations with incident disability among older adults: A population-based study. Age and Ageing, 45(6), 812–819. 10.1093/ageing/afw076 27126329

[bibr19-08982643251395871] Herranen P. Koivunen K. Palviainen T. Kujala U. M. Ripatti S. Kaprio J. Sillanpää E. FinnGen . (2024). Genome-wide polygenic score for muscle strength predicts risk for common diseases and lifespan: A prospective cohort study. The Journals of Gerontology. Series A, Biological Sciences and Medical Sciences, 79(4), glae064. 10.1093/gerona/glae064 PMC10972579 38450701

[bibr20-08982643251395871] Hirvensalo M. Rantanen T. Heikkinen E. (2000). Mobility difficulties and physical activity as predictors of mortality and loss of Independence in the community-living older population. Journal of the American Geriatrics Society, 48(5), 493–498. 10.1111/j.1532-5415.2000.tb04994.x 10811541

[bibr21-08982643251395871] Iso-Markku P. Waller K. Vuoksimaa E. Heikkilä K. Rinne J. Kaprio J. Kujala U. M. (2016). Midlife physical activity and cognition later in life: A prospective twin study. Journal of Alzheimer’s Disease: JAD, 54(4), 1303–1317. 10.3233/JAD-160377 27589524

[bibr22-08982643251395871] Jehu D. A. Skelton D. A. (2023). The measurement and reporting of falls: Recommendations for research and practice on defining faller types. Journal of Frailty, Sarcopenia and Falls, 8(4), 200–203. 10.22540/JFSF-08-200 PMC10690127 38046444

[bibr23-08982643251395871] Kaprio J. Koskenvuo M. (2002). Genetic and environmental factors in complex diseases: The older Finnish twin cohort. Twin Research: The Official Journal of the International Society for Twin Studies, 5(5), 358–365. 10.1375/136905202320906093 12537859

[bibr24-08982643251395871] Koskinen S. Lundqvist A. Ristiluoma N. (2012). Health, functional capacity and welfare in Finland in 2011. National Institute for Health and Welfare (THL). Report 68/2012 pages. 290 Helsinki 2012. https://urn.fi/URN:ISBN:978-952-245-769-1

[bibr25-08982643251395871] Kujala U. M. Kaprio J. Sarna S. Koskenvuo M. (1998). Relationship of leisure-time physical activity and mortality: The Finnish twin cohort. JAMA, 279(6), 440–444. 10.1001/jama.279.6.440 9466636

[bibr26-08982643251395871] Kujala U. M. Leskinen T. Rottensteiner M. Aaltonen S. Ala-Korpela M. Waller K. Kaprio J. (2022). Physical activity and health: Findings from Finnish monozygotic twin pairs discordant for physical activity. Scandinavian Journal of Medicine & Science in Sports, 32(9), 1316–1323. 10.1111/sms.14205 PMC9378553 35770444

[bibr55-08982643251395871] Kwok W. S. Khalatbari-Soltani S. Dolja-Gore X. Byles J. S Oliveira J. Pinheiro M. B. Tiedemann A. Sherrington C. (2024). Falls and patterns of physical activity participation over 18 years in the Australian Longitudinal Study on Women’s Health. British Journal of Sports Medicine, 58(16), 919–929. 10.1136/bjsports-2024-108262 PMC11347968 39048153

[bibr27-08982643251395871] Lafortune L. Martin S. Kelly S. Kuhn I. Remes O. Cowan A. Brayne C. (2016). Behavioural risk factors in mid-life associated with successful ageing, disability, dementia and frailty in later life: A rapid systematic review. PLoS ONE, 11(2), Article e0144405. 10.1371/journal.pone.0144405 PMC4742275 26845035

[bibr28-08982643251395871] Lee J. (2020). The association between physical activity and risk of falling in older adults: A systematic review and meta-analysis of prospective cohort studies. Geriatric Nursing, 41(6), 747–753. 10.1016/j.gerinurse.2020.05.005 32507370

[bibr29-08982643251395871] Malmberg J. J. Miilunpalo S. I. Pasanen M. E. Vuori I. M. Oja P. (2006). Associations of leisure-time physical activity with mobility difficulties among middle-aged and older adults. Journal of Aging and Physical Activity, 14(2), 133–153. 10.1123/japa.14.2.133 19462545

[bibr30-08982643251395871] Mänty M. Heinonen A. Leinonen R. Törmäkangas T. Sakari-Rantala R. Hirvensalo M. von Bonsdorff M. B. Rantanen T. (2007). Construct and predictive validity of a self-reported measure of preclinical mobility limitation. Archives of Physical Medicine and Rehabilitation, 88(9), 1108–1113. 10.1016/j.apmr.2007.06.016 17826454

[bibr31-08982643251395871] McGue M. Osler M. Christensen K. (2010). Causal inference and observational research: The utility of twins. Perspectives on Psychological Science: A Journal of the Association for Psychological Science, 5(5), 546–556. 10.1177/1745691610383511 PMC3094752 21593989

[bibr32-08982643251395871] Morie M. Reid K. F. Miciek R. Lajevardi N. Choong K. Krasnoff J. B. Storer T. W. Fielding R. A. Bhasin S. LeBrasseur N. K. (2010). Habitual physical activity levels are associated with performance in measures of physical function and mobility in older men. Journal of the American Geriatrics Society, 58(9), 1727–1733. 10.1111/j.1532-5415.2010.03012.x PMC2945416 20738436

[bibr33-08982643251395871] Musich S. Wang S. S. Ruiz J. Hawkins K. Wicker E. (2018). The impact of mobility limitations on health outcomes among older adults. Geriatric Nursing, 39(2), 162–169. 10.1016/j.gerinurse.2017.08.002 28866316

[bibr34-08982643251395871] Patel K. V. Coppin A. K. Manini T. M. Lauretani F. Bandinelli S. Ferrucci L. Guralnik J. M. (2006). Midlife physical activity and mobility in older age: The InCHIANTI study. American Journal of Preventive Medicine, 31(3), 217–224. 10.1016/j.amepre.2006.05.005 PMC2646092 16905032

[bibr56-08982643251395871] Pettee Gabriel K. Griswold M. E. Wang W. Conway S. H. Windham B. G. Palta P. Kucharska-Newton A. Pompeii L. A. (2019). Physical activity trajectories and subsequent fall risk: ARIC Study. Preventive Medicine, 121, 40–46. 10.1016/j.ypmed.2019.02.007 PMC6448408 30742870

[bibr35-08982643251395871] Pettee Gabriel K. Sternfeld B. Colvin A. Stewart A. Strotmeyer E. S. Cauley J. A. Dugan S. Karvonen-Gutierrez C. (2017). Physical activity trajectories during midlife and subsequent risk of physical functioning decline in late mid-life: The study of Women’s health across the nation (SWAN). Preventive Medicine, 105, 287–294. 10.1016/j.ypmed.2017.10.005 PMC5873953 28987336

[bibr36-08982643251395871] Piirtola M. Kaprio J. Kujala U. M. Heikkilä K. Koskenvuo M. Svedberg P. Silventoinen K. Ropponen A. (2016). Association between education and future leisure-time physical inactivity: A study of Finnish twins over a 35-year follow-up. BMC Public Health, 16(1), 720. 10.1186/s12889-016-3410-5 PMC4973543 27492437

[bibr37-08982643251395871] Romanov K. Varjonen J. Kaprio J. Koskenvuo M. (2003). Life events and depressiveness – The effect of adjustment for psychosocial factors, somatic health and genetic liability. Acta Psychiatrica Scandinavica, 107(1), 25–33. 10.1034/j.1600-0447.2003.01419.x 12558538

[bibr38-08982643251395871] Sabia S. Singh-Manoux A. Hagger-Johnson G. Cambois E. Brunner E. J. Kivimaki M. (2012). Influence of individual and combined healthy behaviours on successful aging. CMAJ : Canadian Medical Association Journal, 184(18), 1985–1992. 10.1503/cmaj.121080 PMC3519184 23091184

[bibr39-08982643251395871] Schoene D. Heller C. Aung Y. N. Sieber C. C. Kemmler W. Freiberger E. (2019). A systematic review on the influence of fear of falling on quality of life in older people: Is there a role for falls? Clinical Interventions in Aging, 14, 701–719. 10.2147/CIA.S197857 PMC6514257 31190764

[bibr40-08982643251395871] Sherrington C. Fairhall N. Kwok W. Wallbank G. Tiedemann A. Michaleff Z. A. Ng C. A. C. M. Bauman A. (2020). Evidence on physical activity and falls prevention for people aged 65+ years: Systematic review to inform the WHO guidelines on physical activity and sedentary behaviour. The International Journal of Behavioral Nutrition and Physical Activity, 17(1), 144. 10.1186/s12966-020-01041-3 PMC7689963 33239019

[bibr41-08982643251395871] Smith L. Stubbs B. Hamer M. (2019). Changes in physical activity behavior and risk of falls over 8 years’ follow-up: English longitudinal study of aging. Mayo Clinic Proceedings, 94(2), 365–367. 10.1016/j.mayocp.2018.11.014 30711135

[bibr42-08982643251395871] Tikkanen P. Nykanen I. Lonnroos E. Sipila S. Sulkava R. Hartikainen S. (2012). Physical activity at age of 20-64 years and mobility and muscle strength in old age: A community-based study. The Journals of Gerontology. Series A, Biological Sciences and Medical Sciences, 67(8), 905–910. 10.1093/gerona/gls005 22396477

[bibr43-08982643251395871] Tinetti M. E. Kumar C. (2010). The patient who falls: “It’s Always a Trade-off”. JAMA, 303(3), 258–266. 10.1001/jama.2009.2024 PMC3740370 20085954

[bibr44-08982643251395871] Vieira E. R. Palmer R. C. Chaves P. H. M. (2016). Prevention of falls in older people living in the community. BMJ, 353, i1419. 10.1136/bmj.i1419 27125497

[bibr45-08982643251395871] Visser M. Simonsick E. M. Colbert L. H. Brach J. Rubin S. M. Kritchevsky S. B. Newman A. B. Harris T. B. Health A. B. C. (2005). Type and intensity of activity and risk of mobility limitation: The mediating role of muscle parameters. Journal of the American Geriatrics Society, 53(5), 762–770. 10.1111/j.1532-5415.2005.53257.x 15877550

[bibr46-08982643251395871] Waller K. Kaprio J. Kujala U. M. (2008). Associations between long-term physical activity, waist circumference and weight gain: A 30-year longitudinal twin study. International Journal of Obesity, 32(2), 353–361. 10.1038/sj.ijo.0803692 17653065

[bibr47-08982643251395871] Waller K. Kaprio J. Kujala U. M. (2014). Dyspnea and all-cause mortality: 28-yr follow-up study among adult twins. Medicine and Science in Sports and Exercise, 46(8), 1538–1545. 10.1249/MSS.0000000000000255 24500534

[bibr48-08982643251395871] Waller K. Kaprio J. Lehtovirta M. Silventoinen K. Koskenvuo M. Kujala U. M. (2010a). Leisure-time physical activity and type 2 diabetes during a 28 year follow-up in twins. Diabetologia, 53(12), 2531–2537. 10.1007/s00125-010-1875-9 20706830

[bibr49-08982643251395871] Waller K. Kujala U. M. Kaprio J. Koskenvuo M. Rantanen T. (2008). Effect of physical activity on health in twins: A 30-year longitudinal study. Medicine and Science in Sports and Exercise, 42(4), 658–664. 10.1249/mss.0b013e3181bdeea3 19952836

[bibr50-08982643251395871] Waller K. Kujala U. M. Rantanen T. Kauppinen M. Silventoinen K. Koskenvuo M. Kaprio J. (2010b). Physical activity, morbidity and mortality in twins: A 24-year prospective follow-up. European Journal of Epidemiology, 25(10), 731–739. 10.1007/s10654-010-9493-x 20680407

[bibr51-08982643251395871] Waller K. Vähä-Ypyä H. Törmäkangas T. Hautasaari P. Lindgren N. Iso-Markku P. Heikkilä K. Rinne J. Kaprio J. Sievänen H. Kujala U. M. (2018). Long-term leisure-time physical activity and other health habits as predictors of objectively monitored late-life physical activity—A 40-year twin study. Scientific Reports, 8(1), 9400. 10.1038/s41598-018-27704-7 PMC6010475 29925959

[bibr52-08982643251395871] Wang G. Zhou Y. Zhang L. Li J. Liu P. Li Y. Ma L. (2024). Prevalence and incidence of mobility limitation in Chinese older adults: Evidence from the China health and retirement longitudinal study. The Journal of Nutrition, Health & Aging, 28(3), Article 100038. 10.1016/j.jnha.2024.100038 PMC12880464 38280833

[bibr53-08982643251395871] World Health Organization . (2008). WHO global report on falls prevention in older age. Ageing and life course, family and community health: WHO global report on falls prevention in older age. https://iris.who.int/handle/10665/43811

[bibr54-08982643251395871] World Health Organization . (2021). Step safely: Strategies for preventing and managing falls across the life-course. https://www.who.int/publications/i/item/978924002191-4

